# Trends and risk factors of care-seeking delay among students with pulmonary tuberculosis in Wuhan, China, 2012–2025

**DOI:** 10.3389/fpubh.2026.1929226

**Published:** 2026-08-28

**Authors:** Zhengbin Zhang, Wei Zhang, Gang Wu, Jianjie Wang, Aiping Yu, Jun Chen, Zhouqin Lu, Guiyang Wang, Tiantian Wang, Xiaojun Wang, Jing Hu, Yuehua Li

**Affiliations:** 1 Department of Tuberculosis Control and Cure, Wuhan Pulmonary Hospital (Wuhan Institute for Tuberculosis Control), Hubei Branch (Wuhan Pulmonary Hospital) of the National Clinical Research Center for Infectious Diseases, Wuhan, Hubei, China; 2 Infectious Disease Prevention and Control Department, Wuhan Dongxihu Disease Prevention and Control Center, Wuhan, Hubei, China; 3 Occupational Disease Prevention and Control Department, Center for Disease Control and Prevention of Yangtze River Navigation Administration, Wuhan, Hubei, China

**Keywords:** care-seeking delay, COVID-19, pulmonary tuberculosis, risk factor, student

## Abstract

**Background:**

Care-seeking delay among students with tuberculosis (TB) poses a significant barrier to school-based TB prevention and control. Such delay facilitates the transmission of *Mycobacterium tuberculosis* in high-density populations and frequently leads to cluster outbreaks in schools. This retrospective study examined the prevalence, temporal trends, and risk factors of care-seeking delay among student TB patients in Wuhan, China, over a 14-year period (2012–2025).

**Methods:**

This retrospective study used data from the Chinese National Tuberculosis Information Management System (N-TBIMS). All registered student pulmonary tuberculosis (PTB) cases in Wuhan from January 1, 2012, to December 31, 2025, were included. Multivariable logistic regression was used to identify independent risk factors for delay.

**Results:**

A total of 4,920 student PTB patients were enrolled (median age: 19 years, IQR: 17–21,60.54% male). The median time to care-seeking was 9 days (IQR: 3–21), and 37.72% delayed care-seeking for ≥14 days. The delay proportion remained stable at approximately 38.84% during 2012–2019, dropped sharply to 26.55% in 2021, then rebounded to 48.47% in 2022 and reached 55.57% by 2025. Female students experienced a dramatic increase, with rates exceeding 50% during 2022–2025, while male students remained relatively stable. Children aged 3–11 years had the highest delay proportion (55.47%), peaking at nearly 80% in 2023. Multivariable analysis identified key risk factors: non-Han Chinese ethnicity (OR = 2.530, 95% CI: 1.702–3.763, *p* = 0.001), trace detection mode (OR = 2.875, 95% CI: 1.991–4.152, *p* = 0.001), referral detection mode (OR = 1.670, 95% CI: 1.174–2.376, *p* = 0.004), and Sputum bacteria-positive (OR = 0.869,95% CI: 0.764–0.990, *p* = 0.035) compared to Sputum bacteria-negative. Adolescents aged 12–17 years (OR = 0.499,95% CI: 0.333–0.746, *p* = 0.001) and young adults aged 18–22 years (OR = 0.538,95% CI: 0.362–0.801, *p* = 0.002) had approximately 50% lower risk compared to children aged 3–11 years.

**Conclusion:**

Care-seeking delay (≥14 days) affected 37.72% of student TB patients in Wuhan. The COVID-19 pandemic had a dual impact: strict NPIs in 2020–2021 transiently reduced delays, while post-pandemic health-system strain drove a rebound exceeding pre-pandemic levels. Four distinct risk factors for delay emerged: non-Han students, children aged 3–11 years, passively detected cases via contact tracing or referral, and sputum bacteria-positive patients. Targeted health education, school-based symptom screening, expedited diagnosis after referral or tracing, and multisectoral collaboration among schools, families, and TB control centers are urgently needed to reduce delays and avert school-based outbreaks.

## Introduction

Pulmonary tuberculosis (PTB), caused by *Mycobacterium tuberculosis*, remains a major global public health challenge (1). China is one of the 30 high-TB-burden countries, with an estimated 696,000 new cases and an incidence of 49/100,000 in 2024 (2). Within this high-burden context, care-seeking delay among PTB patients extends the pre-diagnosis infectious period, amplifying transmission risk in high-density congregate settings such as schools and workplaces, which can undermine the effectiveness of tuberculosis prevention and control strategies (3, 4). Schools, in particular, represent high-density environments where prolonged infectiousness due to care-seeking delay can rapidly propagate *M. tuberculosis* among susceptible students, precipitating clustered outbreaks that compromise school-based epidemic control efforts. This delay not only undermines outbreak containment but also exacerbates individual health deterioration and adverse social consequences. Prompt care-seeking is therefore essential—not only for interrupting school-based transmission chains but also for mitigating downstream social repercussions, including academic disruption, family economic strain, and community-level stigma (5, 6).

Wuhan, a large metropolitan city and a major educational hub in China, hosts a permanent resident population of approximately 13 million, including over 2.3 million students. In the past decade, Wuhan has reported 5,000–6,500 newly diagnosed PTB cases annually, of which 200–600 were among students, accounting for 4–9% of all notified cases—a proportion consistent with the national average (7, 8). Fifty-four school-based outbreaks were reported in Wuhan between 2017 and 2022, underscoring the vulnerability of congregate educational settings (9, 10). Local epidemiological investigations indicate that care-seeking delay among students with PTB serves as a critical driver of these school-based outbreaks. Although care-seeking delay in the general PTB population has been extensively studied (11–13), research focusing specifically on student populations—and particularly on how contextual disruptions such as the COVID-19 pandemic modify care-seeking behaviors in this group—remains limited.

To address this gap, we analyzed 14-year trends and risk factors of care-seeking delay among students with PTB in Wuhan (2012–2025). This period spans both pre- and post-pandemic phases, enabling assessment of how public health disruptions affect care-seeking patterns. Identifying modifiable risk factors will inform targeted interventions to interrupt school-based TB transmission, we can design specific interventions to prevent further TB spread in schools and reduce the broader health and social damage it causes.

## Methods

### Data collection

The surveillance data for TB in Chinese mainland from 2012 to 2025 were obtained from the National Tuberculosis Information Management System (NTBIMS), which is an internet-based real-time disease-reporting system. The reported cases encompass suspected cases, clinically diagnosed cases and etiologically confirmed cases, which were diagnosed according to the diagnostic criteria issued by the National Health Commission of the People’s Republic of China (NHC). Suspected cases were not included in the analysis after diagnosis and exclusion, focusing on clinically diagnosed and etiologically confirmed cases. The data in the analysis included demographic details (Patient ID, gender, age, nation, region and patient derived source) and clinical diagnosis and treatment details (symptom onset date, diagnosis date and diagnosis category).

### Related definition

#### Pulmonary tuberculosis patients diagnosis

According to the Chinese diagnostic criteria for tuberculosis (WS 288-2017), patients with two or more positive sputum smears, sputum cultures, or Gene-Xpert nucleic acid test results are diagnosed as having pathogen-positive pulmonary tuberculosis. Patients are diagnosed as pathogen-negative if their sputum is found to be negative through laboratory and molecular biology testing, but chest imaging shows lesions consistent with active pulmonary tuberculosis, and either the tuberculin skin test is strongly positive or the interferon-gamma release assay is positive. Patients were categorized into five diagnostic types per Chinese national standards: primary TB, hematogenous disseminated TB, secondary TB, tuberculous pleurisy, and other extrapulmonary TB (14). Extrapulmonary TB was not included, as only PTB cases were mandatorily registered in NTBIMS.

#### Student patients

The subjects are students diagnosed with PTB at designated tuberculosis hospitals in mainland China, ranging from preschoolers to doctoral candidates, including kindergarten, primary school, junior high school, senior high school, university, and graduate students.

#### Days for care-seeking

The time interval between the onset of major symptom appearance date and the first visit to the formal health care facility.

#### Care seeking delay

Care-seeking delay is defined as the time interval between the onset of major TB symptoms and the first visit to a formal healthcare facility. A 14-day cut-off is a commonly used indicator for delay in China’s TB control programs (15).

#### Regional division

In Wuhan’s spatial planning, the region enclosed by the Third Ring Road—designated as the city’s urban development boundary—is treated as the near/central-city zone (Jiang’an, Jianghan, Qiaokou, Hanyang, Wuchang, Qingshan and Hongshan); areas beyond this ring road are categorized as far-city or outer urban districts (Dongxihu, Caidian, Jiangxia, Huangpi, Xinzhou and Hannan).

#### Mode of case detection

Patients were categorized by entry pathway into five groups: direct medical consultation (self-referral without referral), referral (inter-facility transfer from non-designated providers), active screening (systematic population screening), trace (contact investigation), and routine physical examination (incidental detection during health check-ups).

#### Sputum bacteria classification

Patients were grouped as: (i) bacteriologically positive (smear+, culture+, or Xpert+); (ii) bacteriologically negative (all tests negative, clinically diagnosed); or (iii) no bacteriological result (testing not performed). Classification followed WS 288–2017 and WHO 2022 guidelines (16).

#### Therapeutic classification

Patients were stratified into new (no prior or < 1 month of anti-TB treatment) and previously treated/retreatment (≥1 month of prior anti-TB therapy, regardless of outcome) cohorts based on self-reported and medical-record-verified treatment history.

#### Statistical analysis

Data entered into the Statistical Package for Social Science (SPSS) version 25. Descriptive analysis was conducted to present the general epidemiological characteristics of seeking medical care based on demographic information. Continuous variables were reported using the median and interquartile range (IQR), while categorical variables were presented as frequency (n) and percentage (%). The Mann–Whitney test was utilized to compare two groups, while the Kruskal–Wallis test was employed for comparing three or more groups. The influencing factors of delay were analyzed by univariate and multivariate logistic regression models. Adjusted odds ratio (AOR) and corresponding 95% confidence intervals were retrieved. Those variables with *p* < 0.05 were considered as statistically significant differences.

## Results

### Socio demographic characteristics of student patients

A total of 4,920 students with PTB were enrolled in this study, ranging in age from 3 to 36 years, with a median age of 19 (IQR: 17–21) years. The majority of participants were aged 18–22 years (56.26%) and of Han ethnicity (97.84%). Males accounted for 60.54%, while females comprised 39.46%. Regarding spatiotemporal distribution, student patients represented 70.26% (2012–2019), 16.38% (2020–2023), and 13.35% (2024–2025) of the total cases across the three periods, respectively. Geographically, students from near urban areas constituted 71.95% of the cases, compared to 28.05% from far urban areas. Clinically, the vast majority of cases were classified as secondary pulmonary tuberculosis (96.81%), and 99.55% were new (initial treatment) cases. Sputum smear positivity for *M. tuberculosis* was observed in 36.15% of patients. Regarding case detection mode, the primary source was referral (72.03%), followed by trace (20.08%) and direct medical consultation (3.58%) (Table 1).

**Table 1:** Days from onset to first visit among student patients in Wuhan, China, 2012–2025.

Variable	Number of student patients	Time from symptom onset to first seeking care	Mann–Whitney *U* or Kruskal–Wallis *H* value	*p*	Number of delayed first seeking care patients	First seeking care delay proportion	*χ* ^2^	*p*
	*N* (%)	M_d_ (P_25_, P_75_)			*N*	(%)		
Year			38.459	0.001			39.637	0.001
2012–2019	3,457 (70.26%)	9 (3,20)			1,256	36.33%		
2020–2022	806 (16.38%)	8 (3,18)			280	34.74%		
2023–2025	657 (13.35%)	13 (4.5,31)			320	48.71%		
Gender			−2.111	0.035			1.148	0.234
Male	2,979 (60.54%)	9 (3,20)			1,104	37.06%		
Female	1941 (39.46%)	9 (3,23)			752	38.74%		
Age			33.033	0.001			28.816	0.001
3 ~ 11	128 (2.60%)	14.50 (7,34)			71	55.47%		
12 ~ 17	1,422 (28.90%)	9 (3,21)			525	36.92%		
18 ~ 22	2,768 (56.26%)	9 (3,20)			999	36.09%		
23~	602 (12.24%)	11 (4,24)			261	43.36%		
Nation			−4.826	0.001			23.665	0.001
Han Chinese	4,814 (97.84%)	9 (3,21)			1792	37.22%		
Non-Han Chinese	106 (2.16%)	15 (8.25,25)			64	60.38%		
Region			−7.894	0.001			51.321	0.001
Near urban area	3,540 (71.95%)	8 (3,19)			1,226	34.63%		
Far urban area	1,380 (28.05%)	12 (5,27)			630	45.65%		
Mode of case detection			159.921	0.001			102.526	0.001
Direct medical consultation	176 (3.58%)	8 (3,14)			45	25.57%		
Referral	3,544 (72.03%)	8 (3,19)			1,242	35.05%		
Trace	988 (20.08%)	14 (6,32)			504	51.01%		
Physical examination	142 (2.89%)	6 (1,14.25)			39	27.46%		
Proactive screening	70 (1.42%)	9 (2,22)			26	37.14%		
Diagnostic classification			4.743	0.192			2.650	0.448
Primary pulmonary tuberculosis	32 (0.65%)	13.5 (6.5,33)			16	50.00%		
Secondary pulmonary tuberculosis	4,763 (96.81%)	9 (3,21)			1797	37.73%		
Hematogenous disseminated pulmonary tuberculosis	14 (0.28%)	2 (0.75,34.75)			5	35.71%		
Tuberculous pleurisy	111 (2.26%)	9 (4,18)			38	34.23%		
Sputum bacteria classification			−3.013	0.003			6.269	0.012
Sputum bacteria-Positive	1779 (36.15%)	10 (3,24)			712	40.02%		
Sputum bacteria- negative	3,141 (63.85%)	9 (3,20)			1,144	36.42%		
Therapeutic classification			−0.539	0.59			0.095	0.757
Initial treatment	4,898 (99.55%)	9 (3, 21)			1847	37.71%		
Retreatment	22 (0.45%)	7 (1.75,23.50)			9	40.91%		
Total	4,920	9.00 (3.00, 21.00)			1856	37.72%		

### Care-seeking time among student patients

The median care-seeking time of PTB students was 9 days (IQR, 3–21) in Wuhan. Specifically, the care-seeking time of student patients from 2020 to 2023 was significantly shorter compared to the other two time periods (*H* = 38.459, *p*<0.001), The care-seeking time for the 12–17 and 18–22 age groups was significantly shorter than that of the 23 and above age groups, especially shorter than that of the 3–11 age group (*H* = 33.033, *p* < 0.001). Males had longer care-seeking time than females (*Z* = –2.111, *p* = 0.035). Non-Han Chinese student patients exhibited significantly longer care-seeking time than the Han Chinese group (*Z* = –4.826, *p* = 0.001), Similarly, the care-seeking time for student patients residing in far urban areas experienced longer time than those in near urban areas (*Z* = –7.894, *p* < 0.001), Patients with bacteriologically confirmed PTB had significantly longer time than those with clinically diagnosed PTB (*Z* = –3.013, *p* = 0.003). Notably, the student patients from trace had the longest time among the detection modes (*H* = 159.921, *p* < 0.001). Conversely, no statistically significant differences in time of care-seeking were observed regarding diagnostic classification, or therapeutic classification (*p* > 0.05), details of the sociodemographic distribution are provided in Table 1.

### Proportion of care-seeking delay among student patients

The proportion of PTB students who experienced care-seeking delay of ≥14 days in central China was 37.72% (1,856/4,920), and 17.33% (853/4,920) had care-seeking delay of ≥30 days. The proportion of care-seeking delay from 2020 to 2023 (34.74%) decreased compared with the other two time periods (*χ*^2^ = 39.637, *p* < 0.001). The proportion of care-seeking delay in far urban areas (45.65%) was significantly higher than that in near urban areas (34.63%). The proportion of care-seeking delay in the 3–11 age group (55.47%) was significantly higher than that in the other groups (*χ*^2^ = 28.816, *p* < 0.001). Non-Han Chinese student patients had a higher delay proportion than those from the Han Chinese group (*χ*^2^ = 23.665, *p* < 0.001). The delay proportions of student patients from trace (51.01%), proactive screening (37.14%), and referral (35.05%) patient sources were relatively high, and the differences among patient sources were statistically significant (*χ*^2^ = 102.526, *p* < 0.001). The differences in the proportion of care-seeking delay based on gender, diagnostic classification, and therapeutic classification were not statistically significant (*p* > 0.05) (Table 1).

### Temporal trends of care-seeking delay among student patients

**Figure 1:**
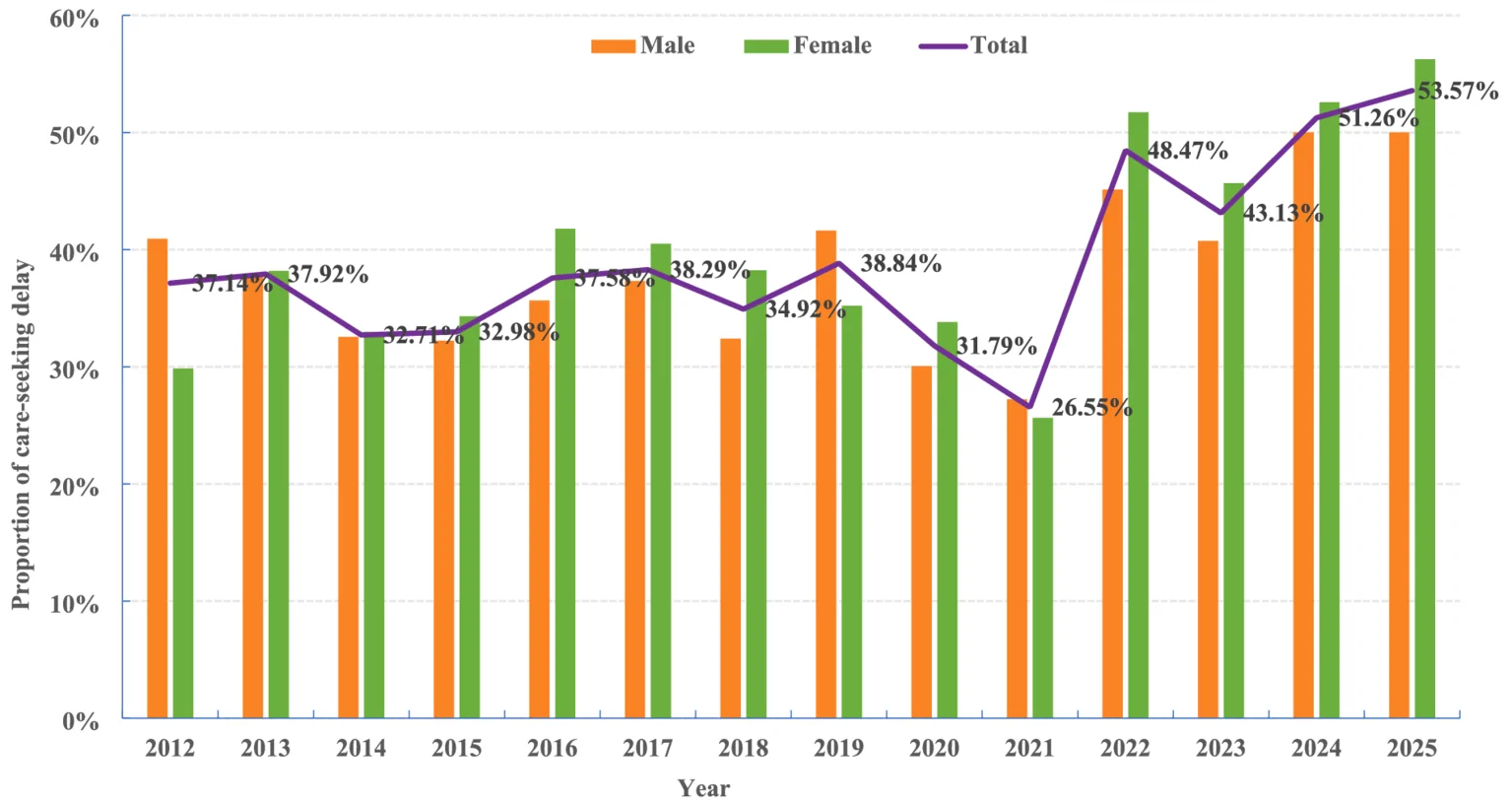
Time distribution on the proportion of care-seeking delay among student patients in Wuhan, China, 2012–2025.

The proportion of student patients who delayed seeking care for ≥14 days showed minimal fluctuation between 2012 and 2019, remaining relatively stable overall. In 2019, the total delay rate stood at 38.84%. However, the rate dropped sharply in 2020 and 2021, reaching a low of 26.55% in 2021. Following this dip, the rate rebounded rapidly: it surged to 48.47% in 2022 and remained elevated at 43.13% in 2023. By 2024 and 2025, the total delay proportion continued to climb, reaching 51.26 and 55.57%, respectively. A gender-disaggregated analysis reveals a stark contrast: Female students (green) experienced a dramatic increase in care-seeking delays, with proportions exceeding 50% during the peak years (2022–2025), significantly higher than any level recorded in the prior decade. In contrast, Male students (orange) showed relative stability throughout the study period, with only minor fluctuations—their delay rates remained consistently below those of female students, peaking at around 50% in 2025 but still lower than their female counterparts (Figure 1).

### Age distribution on proportion of care-seeking delay among student patients

The results indicated that the proportion of student patients delaying care-seeking for ≥14 days varied significantly by age group. Children aged 3–11 years (red line) started at approximately 50% in 2012—the highest among all age groups—and remained relatively high through 2014, during the early COVID-19 containment period (2020–2021), this group experienced a notable decline, reaching a low of ~30% in 2021. However, delay rebounded sharply afterward, peaking at nearly 80% in 2023 before declining again in 2024–2025. Adolescents aged 12–17 years (green line) showed a more moderate trajectory, after an initial rise to ~40% in 2013, delay decreased to a low of ~25% in 2015, then fluctuated between 30 and 45% for most of the period. Notably, in 2023—when delay surged in other age groups—this cohort saw a decline to ~36%, making it the only group to show reduced delay that year.

**Figure 2:**
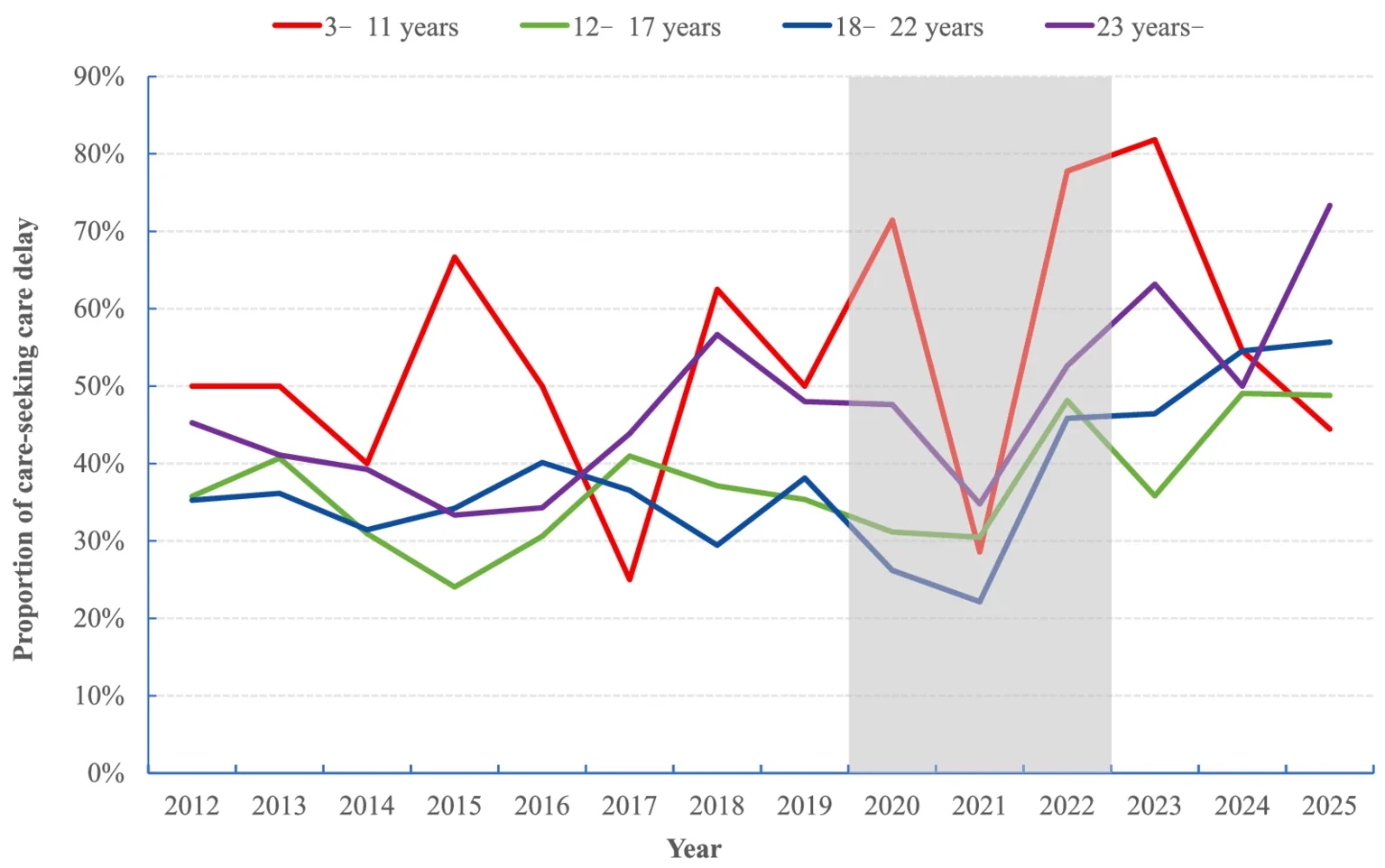
Time-age distribution on the proportion of care-seeking delay among student patients in Wuhan, China, 2012–2025.

Young adults aged 18–22 years (blue line) and those aged ≥23 years (purple line) followed broadly similar trends until 2022, with both groups showing gradual increases and then a dip in 2021. From 2022 onward, however, divergence emerged:

The 18–22 group rose steadily to ~55% by 2025. The 23-and -over group climbed more steeply, surpassing 70% by 2025—the highest among all groups in the final years (Figure 2).

### Factors influencing care-seeking delay among student patients

We conducted a multivariable logistic regression analysis to identify factors associated with care-seeking delay among student tuberculosis patients. The dependent variable was coded as 0 (delay <14 days) or 1 (delay ≥14 days). Independent variables included: year, gender, age group, ethnicity, region, mode of case detection, diagnostic classification, sputum bacteriology status, and treatment history (initial vs. retreatment). After adjusting for confounders and using the 2012–2019 period as the reference category, we found that the risk of delayed care-seeking was slightly reduced during 2020–2022, though not statistically significant (OR = 0.855, 95% CI: 0.720–1.015, *p* = 0.073). In contrast, the 2023–2025 period was associated with a 53.6% significantly higher risk of delay (OR = 1.536, 95% CI: 1.277–1.847, *p* < 0.001). Regarding age, children aged 3–11 years had the highest risk of delayed care. Compared with this group, adolescents aged 12–17 years (OR = 0.499, 95% CI: 0.333–0.746, *p* = 0.001) and young adults aged 18–22 years (OR = 0.538, 95% CI: 0.362–0.801, *p* = 0.002) showed approximately 50% lower risk. For those aged 23-and -over, the risk was also lower but did not reach statistical significance (*p* = 0.196). Non-Han Chinese ethnicity was strongly associated with increased risk: individuals from ethnic minority groups had 2.53 times higher odds of delayed care-seeking compared to Han Chinese (OR = 2.530, 95% CI: 1.702–3.763, *p* < 0.001). No significant association was observed between gender and care-seeking delay (*p* = 0.888). In terms of case detection mode, compared with cases detected through “direct medical consultation,” those detected via “trace” had the highest risk (OR = 2.875, 95% CI: 1.991–4.152, *p* < 0.001), followed by “referral” (OR = 1.670, 95% CI: 1.174–2.376, *p* = 0.004). Cases detected through “physical examination” or “proactive screening” showed no significant difference in risk. These findings highlight that trace and referral systems are key pathways for identifying high-risk cases. Sputum bacteria-negative cases had significantly lower odds of delayed care-seeking compared to sputum bacteria-positive cases (OR = 0.869, 95% CI: 0.764–0.990, *p* = 0.035). Neither diagnostic classification (e.g., primary vs. secondary pulmonary TB) nor treatment history (initial vs. retreatment) showed a statistically significant independent association with care-seeking delay (all *p* > 0.05).

In summary, while several factors—including recent time period (2023–2025), younger age (3–11 years), non-Han ethnicity, and passive case detection via trace or referral—were significantly associated with delayed care-seeking, others such as gender, diagnostic subtype, and treatment history showed no independent effect (Table 2).

**Table 2:** Influencing factors of care-seeking delay among student patients in Wuhan, China.

Independent variable	*β* value	Standard deviation	Wald *χ*^2^	*p-*value	OR (95%*CI*)
Year					
2012–2019					1
2020–2022	−0.157	0.087	3.218	0.073	0.855 (0.720–1.015)
2023–2025	0.429	0.094	20.766	0.001	1.536 (1.277–1.847)
Gender					
Male					1
Female	0.009	0.062	0.02	0.888	1.009 (0.893–1.140)
Age					
3 ~ 11					1
12 ~ 17	−0.696	0.205	11.477	0.001	0.499 (0.333–0.746)
18 ~ 22	−0.620	0.203	9.316	0.002	0.538 (0.362–0.801)
23~	−0.281	0.217	1.670	0.196	0.755 (0.494–1.156)
Nation					
Han Chinese					1
Non-Han Chinese	0.928	0.202	21.027	0.001	2.530 (1.702–3.763)
Region					
Near urban area					
Far urban area	0.456	0.068	44.740	0.001	1.578 (1.381–1.804)
Mode of case detection					
Direct medical consultation					1
Referral	0.513	0.180	8.127	0.004	1.670 (1.174–2.376)
Trace	1.056	0.187	31.751	0.001	2.875 (1.991–4.152)
Physical examination	0.123	0.260	0.223	0.636	1.131 (0.680–1.880)
Proactive screening	0.305	0.312	0.959	0.327	1.357 (0.737–2.500)
Diagnostic classification					
Primary pulmonary tuberculosis					1
Secondary pulmonary tuberculosis	0.200	0.398	0.253	0.615	1.222 (0.560–2.666)
Hematogenous disseminated pulmonary tuberculosis	−0.017	0.697	0.001	0.980	0.983 (0.251–3.857)
Tuberculous pleurisy	−0.113	0.442	0.065	0.798	0.893 (0.376–2.123)
Sputum bacteria classification					
Sputum bacteria-positive					1
Sputum bacteria-negative	−0.140	0.066	4.467	0.035	0.869 (0.764–0.990)
Therapeutic classification					
Initial treatment					1
Retreatment	0.071	0.445	0.025	0.874	1.073 (0.449–2.567)
Constant	−2.237	0.552	16.438	0.001	0.107

## Discussion

This study provides a comprehensive analysis of care-seeking delay among student tuberculosis (TB) patients in Wuhan, China, based on 4,920 pulmonary TB cases over a 14-year period (2012–2025). Our findings reveal that 37.72% of students delayed care-seeking for ≥14 days, with a median delay of 9 days (IQR: 3–21). While this is shorter than many international settings—such as Ethiopia (35 days) (17), India (16 days) (18), Portugal (37 days) (19), and Myanmar (21 days) (20)—it remains a significant concern given the high-density school environment and the potential for cluster outbreaks (5, 7, 9).

### Temporal trends and the dual impact of COVID-19

As shown in Figure 1, the proportion of care-seeking delay remained relatively stable between 2012 and 2019 (around 38.84% in 2019). A sharp decline occurred in 2020–2021, reaching a low of 26.55% in 2021 which was the lowest point in the entire period. This decline is likely attributable to the stringent non-pharmaceutical interventions (NPIs) implemented during the COVID-19 pandemic, including daily symptom monitoring, universal nucleic acid testing, and mandatory reporting of febrile illnesses. These measures inadvertently facilitated earlier detection of TB symptoms, as individuals with cough or fever were more likely to be identified and referred for chest radiography (21–23). However, following this decline, a dramatic rebound occurred. The delay rate surged to 48.47% in 2022 and remained elevated at 43.13% in 2023, before climbing further to 51.26 and 55.57% in 2024 and 2025, respectively—exceeding 50% for the first time in the observed period. This resurgence can be attributed to several interconnected factors: (1) the lagged effect of healthcare system disruption and resource reallocation during the pandemic, which created a backlog of undiagnosed cases (24, 25); (2) pandemic fatigue and altered risk perception, leading individuals to normalize mild respiratory symptoms and resort to self-medication (26); and (3) the rapid return of population mobility and social interactions after the lifting of restrictions, which facilitated TB transmission (27).

Notably, gender-disaggregated analysis (Figure 1) revealed a stark contrast: female students experienced a dramatic increase in delays, with proportions exceeding 50% during 2022–2025, while male students showed relative stability throughout the study period. This gender disparity warrants further investigation into gender-specific barriers or perceptions that may have been amplified during the post-pandemic period.

### Age-related patterns of care-seeking delay

Figure 2 illustrates the age-specific trends in care-seeking delay. Children aged 3–11 years consistently had the highest delay rates, starting at approximately 50% in 2012 and peaking at nearly 80% in 2023. Three factors likely explain this pattern. First, low perceived risk among parents: given universal BCG vaccination in China, the incidence of TB among young children is relatively low, which may diminish parental vigilance (12, 28). Second, young children have limited ability to articulate symptoms, placing the burden of recognition entirely on parents or teachers, who may not associate vague symptoms such as malaise or poor appetite with TB (13, 29). Third, the diagnostic pathway for pediatric TB is inherently complex, often requiring multiple visits for confirmation, which may indirectly prolong the decision to seek care (29). Interestingly, adolescents aged 12–17 years behaved differently from other age groups. As shown in Figure 2, they were the only cohort to show a decline in delay in 2023 (to approximately 36%), when delays surged in all other groups. This may reflect greater health awareness through school-based education or peer networks. In contrast, older students (≥18 years) showed accelerating delays after 2022, with the ≥23 group surpassing 70% by 2025—the highest among all groups in the final years. This suggests that emerging adulthood, marked by transitions to employment, independent living, or geographic mobility, may introduce new barriers to timely care-seeking. Students in this age group often face the dual pressures of job hunting and postgraduate exam preparation. The resulting time constraints, high stress, and competing priorities may cause them to overlook early symptoms or endure illness without seeking care, thereby prolonging delays.

### Multi-variable analysis of risk factors

The multi-variable logistic regression analysis (Table 2) identified several independent risk factors for care-seeking delay. Consistent with the bivariable analysis, non-Han Chinese ethnicity was strongly associated with increased risk (OR = 2.530, 95% CI: 1.702–3.763, *p* < 0.001). This disparity may stem from weaker awareness of TB prevention and control among ethnic minorities, language barriers—particularly among international students—fear of school suspension or repatriation, and potential influence of religious beliefs (9, 30). Such concerns may drive students to self-medicate through pharmacies or online sources, further delaying timely detection. Children aged 3–11 years constituted the highest-risk age group, with adolescents aged 12–17 (OR = 0.499,95% CI: 0.333–0.746, *p* = 0.001) and young adults aged 18–22 (OR = 0.538,95% CI: 0.362–0.801, *p* = 0.002) showing approximately 50% lower risk. This reinforces the need for targeted interventions for the youngest students and their parents. Regarding case detection mode, students identified through trace had the highest risk of delay (OR = 2.875, 95% CI: 1.991–4.152, *p* < 0.001), followed by those identified through referral (OR = 1.670, 95% CI: 1.174–2.376, *p* = 0.004). This counter intuitive finding—that cases detected through active surveillance mechanisms actually had longer delay—suggests that trace and referral often capture cases that have already been symptomatic for extended periods. In other words, these mechanisms are identifying cases that earlier detection systems failed to catch. This underscores the need to pair trace with accelerated diagnostic confirmation and immediate linkage to care (1). Sputum bacteria-negative cases had 13.1% lower odds of delay than positive cases (OR = 0.869, 95%CI 0.764–0.990, *p* = 0.035). This may reflect a self-reinforcing cycle: delayed care-seeking allows TB bacteria to proliferate, increasing the likelihood of positive sputum results at the time of diagnosis. Consistent with the descriptive analysis (Table 1), gender, diagnostic classification, and treatment history showed no independent association with delay in the multi-variable model (all *p* > 0.05), suggesting that these factors do not independently influence care-seeking behavior within this student population.

### Public health implications

The finding that 37.72% of students delayed care-seeking ≥ 14 days—with rates exceeding 50% in recent years—has serious implications for school-based TB control. Delayed care-seeking prolongs infectiousness, increases the risk of transmission in high-density settings, and contributes to cluster outbreaks. The post-pandemic resurgence, particularly among female students and older age groups, signals an urgent need to reinvigorate school-based TB surveillance and health promotion.

Based on our findings, we recommend three priority actions. First, culturally tailored health education should be developed for ethnic minority and international students, addressing language barriers, stigma, and fear of academic disruption. Second, school-based active case finding should be strengthened through daily symptom screening, cause-of-absence tracking, and streamlined referral pathways. Third, a collaborative school–family–disease control center partnership should be formalized to ensure timely information sharing, joint follow-up for symptomatic students, and continuous supervision of TB control measures.

### Strengths and limitations

This study has several strengths. To our knowledge, it is among the first to specifically examine care-seeking delay among student PTB patients over a 14-year period spanning both pre-pandemic and post-pandemic phases. The use of nationally mandated surveillance data from NTBIMS ensured a large sample size representative of Wuhan’s student population. Several limitations should be acknowledged. First, we were unable to assess individual-level determinants—such as family income, health insurance status, TB-related health literacy, accessibility of local medical resources, or religious beliefs which may independently influence care-seeking behavior. Second, student PTB cases who sought care and were registered outside Wuhan but were enrolled in Wuhan schools were not captured, which may limit the generalizability of our findings. Third, educational level—a potentially relevant stratifier—was incompletely recorded, precluding stratification by school stage and potentially introducing confounding. Fourth, because data were extracted from a routine surveillance system rather than a purpose-designed questionnaire, misclassification or information bias cannot be ruled out (31).

Future studies should integrate qualitative methods to explore the underlying reasons for care-seeking delay and evaluate the real-world effectiveness of targeted interventions.

## Conclusion

This 14-year study found that care-seeking delay (≥14 days) remained common among student PTB patients in Wuhan, affecting 37.72% of cases (median: 9 days). Four distinct risk factors emerged: non-Han Chinese students (OR = 2.530), children aged 3–11 years (55.47% delay), cases detected via contact tracing (OR = 2.875) or referral (OR = 1.670), and sputum bacteria-positive patients (higher delay risk; reference: positive). The COVID-19 pandemic had a dual effect: stringent NPIs in 2020–2021 transiently lowered delays, while post-pandemic health-system strain drove a resurgence exceeding 50% by 2025. Mitigating delay requires culturally tailored health education, school-based symptom screening, expedited diagnosis after trace/referral, and a coordinated school—family—TB control partnership.

## Data Availability

The original contributions presented in the study are included in the article/Supplementary material, further inquiries can be directed to the corresponding author.
